# The impact of engaging leadership on employee engagement and team effectiveness: A longitudinal, multi-level study on the mediating role of personal- and team resources

**DOI:** 10.1371/journal.pone.0269433

**Published:** 2022-06-29

**Authors:** Greta Mazzetti, Wilmar B. Schaufeli

**Affiliations:** 1 Department of Education Studies, University of Bologna, Bologna, Italy; 2 Research Unit Occupational & Organizational Psychology and Professional Learning, KU Leuven, Belgium; 3 Department of Psychology, Utrecht University, Utrecht, The Netherlands; Mugla Sitki Kocman University: Mugla Sitki Kocman Universitesi, TURKEY

## Abstract

Most research on the effect of leadership behavior on employees’ well-being and organizational outcomes is based on leadership frameworks that are not rooted in sound psychological theories of motivation and are limited to either an individual or organizational levels of analysis. The current paper investigates whether individual and team resources explain the impact of engaging leadership on work engagement and team effectiveness, respectively. Data were collected at two time points on *N* = 1,048 employees nested within 90 work teams. The Multilevel Structural Equation Modeling results revealed that personal resources (i.e., optimism, resiliency, self-efficacy, and flexibility) partially mediated the impact of T1 individual perceptions of engaging leadership on T2 work engagement. Furthermore, joint perceptions of engaging leadership among team members at T1 resulted in greater team effectiveness at T2. This association was fully mediated by team resources (i.e., performance feedback, trust in management, communication, and participation in decision-making). Moreover, team resources had a significant cross-level effect on individual levels of engagement. In practical terms, training and supporting leaders who inspire, strengthen, and connect their subordinates could significantly improve employees’ motivation and involvement and enable teams to pursue their common goals successfully.

## Introduction

Multiple studies suggest that work engagement, which is defined as a positive, work-related state of mind characterized by vigor, dedication, and absorption [1], is related to extremely positive outcomes, particularly in terms of employees’ well-being and job performance (for a narrative overview see [2]; for a meta-analysis see [3]).

Therefore, when work engagement is arguably beneficial for employees and organizations alike, the million-dollar question (quite literally, by the way) is: how can work engagement be increased? Schaufeli [4] has argued that operational leadership is critical for enhancing follower’s work engagement. Based on the logic of the Job Demands-Resources (JD-R) model [5], he reasoned that team leaders may (or may not) monitor, manage, and allocate job demands and resources to increase their follower’s levels of work engagement. In doing so, team leaders boost the motivational process that is postulated in the JD-R model. This process assumes that job resources and challenging job demands are inherently motivating and will lead to a positive, affective-motivational state of fulfillment in employees known as work engagement.

The current study focuses on a specific leadership style, dubbed engaging leadership and rooted in Self-Determination Theory (SDT) [6]. Engaging leaders inspire, strengthen, and connect their followers, thereby satisfying their basic psychological needs of autonomy, competence, and relatedness, respectively. In line with the motivational process of the JD-R model, cross-sectional evidence suggests that engaging leaders increase job resources [7] and personal resources [8], which, in their turn, are positively associated with work engagement. So far, the evidence for this mediation is exclusively based on cross-sectional studies. Hence, the first objective of our paper is to confirm the mediation effect of resources using a longitudinal design.

Scholars have emphasized that “the study of leadership is inherently multilevel in nature” (p. 4) [9]. This statement implies that, in addition to the individual level, the team level of analysis should also be included when investigating the impact of engaging leadership.

**Fig 1:**
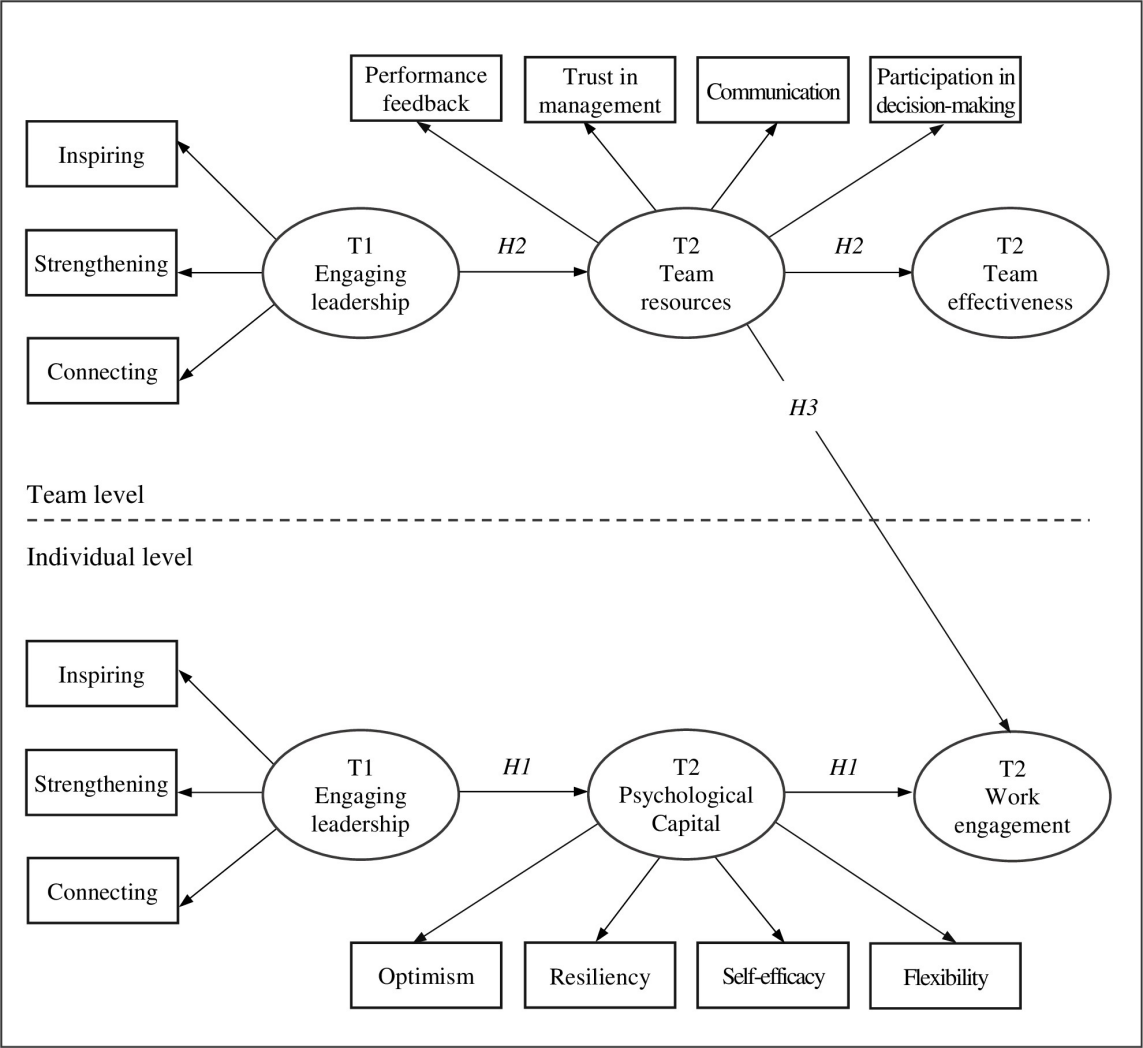
The hypothesized multilevel model of engaging leadership.

The current study makes two notable contributions to the literature. First, it investigates the impact, over time, of a novel, specific leadership style (i.e., engaging leadership) on team- and individual outcomes (i.e., team effectiveness and work engagement). Second, it investigates the mediating role of team resources and personal resources in an attempt to explain the impact of leadership on these outcomes. The research model, which is described in greater detail below, is displayed in Fig 1.

### Leadership and work engagement

Leadership is defined as the way in which particular individuals–leaders–purposefully influence other individuals–their followers–to obtain defined outcomes [10].

A systematic narrative review identified twenty articles on leadership and work engagement [11] and showed that work engagement is positively associated with various person-centered leadership styles. The most pervasively used framework was transformational leadership, whereas authentic, ethical, and charismatic leadership was used much less. The authors conclude that "most of the reviewed studies were consistent in arguing that leadership is significantly correlated with and is affecting employee’s work engagement directly or via mediation” (p. 18) [11]. Moreover, they also conclude that research findings and inferences on leadership and engagement remain narrowly focused and inconclusive due to the lack of longitudinal designs addressing this issue. A recent meta-analysis [12] identified 69 studies and found substantial positive relationships of work engagement with ethical (k = 9; ρ = .58), transformational (k = 36; ρ = .46) and servant leadership (k = 3; ρ = .43), and somewhat less strong associations with authentic (k = 17; ρ = .38) and empowering leadership (k = 4; ρ = .35). Besides, job resources (e.g., job autonomy, social support), organizational resources (e.g., organizational identification, trust), and personal resources (self-efficacy, creativity) mediated the effect of leadership on work engagement. Although transformational leadership is arguably the most popular leadership concept of the last decades [13], the validity of its conceptual definition has been heavily criticized, even to the extent that some authors suggest getting “back to the drawing board” [14]. It should be noted that three main criticisms are voiced: (1) the theoretical definition of the transformational leadership dimensions is meager (i.e., how are the four dimensions selected and how do they combine?); (2) no causal model is specified (i.e., how is each dimension related to mediating processes and outcomes?); (3) the most frequently used measurement tools are invalid (i.e., they fail to reproduce the dimensional structure and do not show empirical distinctiveness from other leadership concepts). Hence, it could be argued that the transformational leadership framework is not very well suited for exploring the impact of leadership on work engagement.

Schaufeli [7] introduced the concept of *engaging leadership*, which is firmly rooted in Self-Determination Theory. According to Deci and Ryan [6], three innate psychological needs are essential ‘nutrients’ for individuals to function optimally, also at the workplace: the needs for *autonomy* (i.e., feeling in control), *competence* (i.e., feeling effective), and *relatedness* (i.e., feeling loved and cared for). Moreover, SDT posits that employees are likely to be engaged (i.e., internalize their tasks and show high degrees of energy, concentration, and persistence) to the degree that their needs for autonomy, competence, and relatedness are satisfied [15]. This is in line with Bormann and Rowold [16]. Based on a systematic review on construct proliferation in leadership research, these authors recommended that leadership concepts should use SDT because this motivational theory allows a more parsimonious description of the mechanisms underlying leadership behaviors. These authors posited that the core of "narrow" leadership constructs "bases on a single pillar" (p. 163), and therefore predict narrow outcomes. In contrast to broad leadership constructs, the concept of engaging leadership is *narrow* because it focuses on leadership behaviors to explicitly promote work engagement.

Schaufeli [7] reasoned that leaders, who are instrumental in satisfying their followers’ basic needs, are likely to increase their engagement levels. More specifically, engaging leaders are supposed to: (1) *inspire* (e.g., by enthusing their followers for their vision and plans, and by making them feel that they contribute to something important); (2) *strengthen* (e.g., by granting their followers freedom and responsibility, and by delegating tasks); and (3) *connect* (e.g., by encouraging collaboration and by promoting a high team spirit among their followers). Hence, by inspiring, strengthening, and connecting their followers, leaders stimulate the fulfillment of their follower’s basic psychological needs for autonomy, competence, and relatedness, respectively, which, in turn, will foster work engagement.

The underlying mechanisms of the relationship between engaging leadership and work engagement are a major focus of research, as the construct of engaging leadership was built upon the identification of the leadership behaviors that are capable of stimulating positive outcomes by satisfying needs. The literature on engaging leadership provides empirical evidence for its indirect impact on followers’ engagement by fulfilling followers’ basic needs. This finding is consistent across occupational sectors and cultural contexts [17–19]. Further, the observation of a partial mediation effect for need satisfaction suggests the presence of a direct relationship between engaging leadership and engagement [20,21]. In their behaviors, engaging leaders are likely to improve their job characteristics to the point of stimulating greater engagement among their employees. This assumption has been corroborated by a recent longitudinal study that delved deeper into the association between engaging leadership and needs satisfaction [22]. That study found that the relationship between engaging leadership and basic needs satisfaction is mediated by enhanced levels of job resources (among them were improved feedback and skill use and better person-job fit). The fulfilment of those needs, in turn, resulted in higher levels of work engagement. Therefore, perceived job resources seem to play a crucial role in the causal relationship between engaging leadership and basic needs satisfaction. This evidence found support in a later two-wave full panel design with a 1-year time lag, where engaging leadership promoted employees’ perception of autonomy and social support from colleagues [23]. In addition, a recent study by Van Tuin and colleagues [24] revealed that engaging leadership is associated with increased perceptions of intrinsic organizational values (e.g., providing a contribution to organizational and personal development) and satisfaction of the need for autonomy which, in turn, may boost employees’ level of engagement.

A recent study investigated the ways in which engaging leadership could boost the effects of human resource (HR) practices for promoting employees’ psychological, physical, and social well-being over time [25]. Teams led by an engaging leader reported higher levels of happiness at work and trust in leadership, combined with lower levels of burnout than their colleagues who were led by poorly engaging leaders. Happiness and trust played a key role in improving team member performance. These findings indicate that engaged leaders provide a thoughtful implementation of HR practices focused on promoting employee well-being, being constantly driven by their employees’ flourishing.

Another line of studies may reveal the causality between engaging leadership and work-related outcomes. A multilevel longitudinal study provided cross-level and team-level effects of engaging leadership [26]. Engaging leadership at T1 explained team learning, innovation, and individual performance through increased teamwork engagement at T2. Interventions targeting engaging leadership created positive work outcomes for leaders (e.g., autonomy satisfaction and intrinsic motivation) and decreased employee absenteeism [27]. However, cross-lagged longitudinal analyses indicate that employees’ current level of work engagement predicts their leaders’ level of engaging leadership rather than the other way around [23]. These findings imply that the relationship between engaging leadership and work engagement cannot be narrowed to a simple unidirectional causal relationship but rather exhibits a dynamic nature, where engaging leadership and work engagement mutually influence each other. The dynamic nature of engaging leadership has also been investigated through a diary study. The results suggest that employees enacted job crafting strategies more frequently on days when leaders were more successful in satisfying their need for connectivity [28]. Hence, leaders who satisfy the need for connectedness among their followers will not only encourage higher levels of engagement among their followers but also an increased ability to proactively adapt tasks to their interests and preferences.

Since transformational leadership is currently the most frequently studied leadership style, a summary of the similarities and differences in the proposed new conceptualization of leadership proposed (i.e., engaging leadership) must be provided.

A key difference between transformational and engaging leadership originates from their foundation. Whereas transformational leadership is primarily a change-oriented style, engaging leadership encourages employees’ well-being through the promotion of supportive relationships and is defined as a relationship-oriented leadership style [29].

Further similarities entail the combination of behaviors meant to foster employees’ well-being and growth. Transformational leaders act as role models admired and emulated by followers (idealized influence), encourage a reconsideration of prevailing assumptions and work practices to promote stronger innovation (intellectual stimulation), identify and build on the unique characteristics and strengths of each follower (individualized consideration), and provides a stimulating view of the future and meaning of their work (inspirational motivation) [30]. A considerable resemblance involves the dimensions of inspirational motivation and inspiring, which are, respectively, included in transformational and engaging leadership. They both entail recognizing the leader as a guiding light to a specific mission and vision, where individual inputs are credited as essential ingredients in achieving the shared goal. Thus, they both fulfill the individual need for meaningfulness. In a similar vein, transformational and engaged leaders are both committed to promote followers’ growth in terms of innovation and creativity. In other words, the intellectual stimulation offered by transformational leadership and the strengthening component of engaging leadership are both aimed at meeting the need for competence among followers.

Alternatively, it is also possible to detect decisive differences between the dimensions underlying these leadership styles. Transformational leadership entails the provision of personal mentorship (i.e., individualized consideration), while engaging leadership is primarily focused on enhancing the interdependence and cohesion among team members (i.e., team consideration). Furthermore, engaging leadership disregards the notion of idealized influence covered by transformational leadership: an engaging leader is not merely identified as a model whose behavior is admired and mirrored, but rather proactively meets followers’ need for autonomy through the allocation of tasks and responsibilities.

Empirical results lent further support to the distinctiveness between transformational and engaging leadership. The analysis of the factor structure of both constructs revealed that measures of engaging and transformational leadership load on separate dimensions instead of being explained by a single latent factor [31]. More recently, additional research findings pointed out that engaging and transformational leadership independently account for comparable portions of variance in work engagement [32]. However, this does not alter the fact that a certain overlap exists between both leadership concepts; thus, it is not surprising that a consistent, positive relationship is found between transformational leadership and work engagement [11].

In sum: a positive link appears to exist between person-centered leadership styles and work engagement. Moreover, this relationship seems to be mediated by (job and personal) resources. However, virtually all studies used cross-sectional designs, and the causal direction remains unclear. We followed the call to go back to the drawing board by choosing an alternative, deductive approach by introducing the theory-grounded concept of engaging leadership and investigate its impact on individual and team outcomes (see Fig 1).

### Engaging leadership, personal resources, and employee engagement (individual level)

Serrano and Reichard [33], who posit that leaders may pursue four pathways to increase their follower’s work engagement: (1) design meaningful and motivating work; (2) support and coach their employees; (3**)** facilitate rewarding and supportive coworker relations, and (4) enhancing personal resources. In the present study, we focus on the fourth pathway. Accordingly, a cross-sectional study using structural equation modeling [8] showed that psychological capital (i.e., self-efficacy, optimism, resiliency, and flexibility) fully mediated the relationship between perceived engaging leadership and follower’s work engagement. Consistent with findings on job resources, this study indicated that personal resources also mediate the relationship between engaging leadership and work engagement. In a nutshel, when employees feel autonomous, competent, and connected to their colleagues, their own personal resources benefit, and this fuels their level of engagement.

In the current study, we use the same conceptualization of psychological capital (PsyCap) as Schaufeli [7,8], which slightly differs from the original concept. Originally, PsyCap was defined as a higher-order construct that is based on the shared commonalities of four first-order personal resources: “(1) having confidence (self-efficacy) to take on and put in the necessary effort to succeed at challenging tasks; (2) making a positive attribution (optimism) about succeeding now and in the future; (3) persevering toward goals and, when necessary, redirecting paths to goals (hope) to succeed; and (4) when beset by problems and adversity, sustaining and bouncing back and even beyond (resiliency) to attain success” (p. 10) [34]. Instead of hope, flexibility is included; that is, the capability of employees to adapt to new, different, and changing requirements at work. Previous research showed a high correlation (*r* > .70) between hope and optimism, thus increasing the risk of multicollinearity [35]. This strong relationship points at conceptual overlap: hope is defined as the perception that goals can be set and achieved, whereas optimism is the belief that one will experience good outcomes. Hence, trust in achieving goals (hope) implies optimism. Additionally, hope includes "when necessary, redirecting paths to goals", which refers to flexibility. Finally, in organizational practice, the flexibility of employees is considered an essential resource because organizations are continuously changing, which requires permanent adaption and hence employee flexibility. In short, there are psychometric, conceptual, and pragmatic arguments for replacing hope by flexibility.

According to Luthans and colleagues [36], PsyCap is a state-like resource representing an employee’s motivational propensity and perseverance towards goals. PsyCap is malleable and open to development, thus it can be enhanced through positive leadership [37]. Indeed, it was found that transformational leadership enhances PsyCap, which, in turn, increases in-role performance and organizational citizenship behavior [38]. In a similar vein, PsyCap mediates the relationships between authentic leadership and employee’s creative behavior [39].

We argue that engaging leadership may promote PsyCap as well. After all, by *inspiring* followers with a clear, powerful and compelling vision, engaging leaders: (1) create the belief in their ability to perform tasks that tie in with that vision successfully, thereby fostering follower’s *self-efficacy*; (2) generate a positive appraisal of the future, thereby fostering follower’s *optimism*; (3) trigger the ability to bounce back from adversity because a favorable future is within reach, thereby fostering follower’s *resiliency*; (4) set goals and induce the belief that these can be achieved, if necessary by redirecting paths to those goals, thereby fostering follower’s *flexibility* [38].

Furthermore, engaging leaders *strengthen* their followers and unleash their potential by setting challenging goals. This helps to build followers’ confidence in task-specific skills, thereby increasing their *self-efficacy* levels, mainly via mastery experiences that occur after challenging goals have been achieved [40]. Setting high-performance expectations also elevates follower’s sense of self-worth, thereby leading to a positive appraisal of their current and future circumstances (i.e., *optimism*). Moreover, a strengthening leader acts as a powerful contextual resource that augments followers’ self-confidence and, hence, increases their ability to bounce back from adversity (i.e., *resiliency*) and adapt to changing requirements at work (i.e., *flexibility*).

Finally, by *connecting* their followers, engaging leaders promote good interpersonal relationships and build a supportive team climate characterized by collaboration and psychological safety. Connecting leaders also foster commitment to team goals by inducing a sense of purpose, which energizes team members to contribute toward the same, shared goal. This means that in tightly knit, supportive and collaborative teams, followers: (1) experience positive emotions when team goals are met, which, in turn, fosters their level of *self-efficacy* [40]; (2) feel valued and acknowledged by others, which increases their self-worth and promotes a positive and *optimistic* outlook; (3) can draw upon their colleagues for help and support, which enables to face problems and adversities with *resiliency*; (4) can use the abilities, skills, and knowledge of their teammates to adapt to changing job and team requirements (i.e., *flexibility*).

In sum, when perceived as such by followers, engaging leadership acts as a sturdy contextual condition that enhances their PsyCap. We continue to argue that, in its turn, high levels of PsyCap are predictive for work engagement; or in other words, PsyCap mediates the relationship between engaging leadership and work engagement.

How to explain the relationship between PsyCap and work engagement? Sweetman and Luthans [41] presented a conceptual model, which relates PsyCap to work engagement through positive emotions. They argue that all four elements of PsyCap may have a direct and state-like relationship with each of the three dimensions of work engagement (vigor, dedication, and absorption). Furthermore, an upward spiral of PsyCap and work engagement may be a source of positive emotion and subsequently broaden an employee’s growth mindset, leading to higher energy and engagement [42,43]. In short, PsyCap prompts and maintains a motivational process that leads to higher work engagement and may ultimately result in positive outcomes, such as job satisfaction and organizational commitment [44].

Psychological capital is a valuable resource to individuals [45] that fosters work engagement, as demonstrated in past research [46]. Hence, following the reasoning above, we formulate the following hypothesis:

Hypothesis 1: *Psychological capital (self-efficacy*, *optimism*, *resiliency*, *and flexibility) mediates the relationship between T1 employee’s perceptions of engaging leadership and T2 work engagement*.

### Engaging leadership, team resources, and work team effectiveness (team level)

So far, we focused on individual-level mediation, but an equivalent mediation process is expected at the aggregated team level as well. We assume that leaders display a comparable leadership style toward the entire team, resulting in a similar relationship with each of the team members. This model of leader-follower interactions is known as the average leadership style (ALS) [47]. This means that homogeneous leader-follower interactions exist *within* teams, but relationships of leaders with followers may differ *between* teams. The relationships between leadership and team effectiveness might be based on an analogous, team-based ALS-approach as well [48]. Following this lead, we posit that team members share their perceptions of engaging leadership, while this shared perception differs across teams. Moreover, we assume that these shared perceptions are positively related to team effectiveness.

An essential role for leaders is to build team resources, which motivate team members and enable them to perform. Indeed, the influence of leader behaviors on team mediators and outcomes has been extensively documented [49,50].

Most studies use the heuristic input-process-output (IPO) framework [51] to explain the relationship between leadership (input) and team effectiveness (output), whereby the intermediate processes describe how team inputs are transformed into outputs. It is widely acknowledged that two types of team processes play a significant role: “taskwork” (i.e., functions that team members must perform to achieve the team’s task) and “teamwork” (i.e., the interaction between team members, necessary to achieve the team’s task). Taskwork is encouraged by task-oriented leadership behaviors that focus on task accomplishment. In contrast, teamwork is encouraged by person-oriented leadership behaviors that focus on developing team members and promoting interactions between them [49]. The current paper focuses on teamwork and person-oriented (i.e., engaging) leadership.

Collectively, team resources such as performance feedback, trust in management, communication between team members, and participation in decision-making constitute a supportive team climate that is conducive for employee growth and development, and hence fosters team effectiveness, as well as individual work engagement. This also meshes with Serrano and Reichard [33], who argue that for employees to flourish, leaders should design meaningful and motivating work (e.g., through feedback and participation in decision making) and facilitate rewarding and supportive coworker relations (e.g., through communication and trust in management).

To date, engaging leadership has not been studied at the team level and concerning team resources and team effectiveness. How should the association between engaging leadership and team resources be conceived? By strengthening, engaging leaders provide their team members with performance feedback; by inspiring, they grant their team members participate in decision making; and by connecting, they foster communication between team members and install trust. Please note that team resources refer to shared, individual perceptions of team members, which are indicated by within-team consensus. Therefore, taken as a whole, the team-level resources that are included in the present study constitute a supportive team climate that is characterized by receiving feedback, trust in management, communication amongst team members, and participating in decision-making. We have seen above that engaging leaders foster team resources, but how are these resources, in their turn, related to team effectiveness?

The multi-goal, multi-level model of feedback effects of DeShon and colleagues [52] posits that individual and team regulatory processes govern the allocation of effort invested in achieving individual and team goals, resulting in individual and team effectiveness. We posit that the shared experience of receiving the team leader’s feedback prompts team members to invest efforts in achieving team tasks, presumably through team regulatory processes, as postulated in the multi-goal, multi-level model.

Trust has been defined as: “the willingness of a party to be vulnerable to the actions of another party based on the expectation that the other party will perform a particular action to the trustor, irrespective of the ability to monitor or control that other party” (p. 712) [53]. Using a multilevel mediation model, Braun and colleagues [54] showed that trust mediates the relationship between transformational leadership and performance at the team level. They reasoned that transformational leaders take into account a team member’s needs, goals, and interests, making them more willing to be vulnerable to their supervisor. This would apply even more for engaging leaders, which is *defined* in terms of satisfying basic follower’s needs. It is plausible that a team’s shared trust in its leader enhances the trust of team members in each other. That means that team members interact and communicate trustfully and rely on each other’s abilities, which, in turn, is conducive for team effectiveness [55].

Communication is a crucial element of effective teamwork [56]. Team members must exchange information to ascertain other members’ competence and intentions, and they must engage in communication to develop a strategy and plan their work. Several studies have shown that effectively gathering and exchanging information is essential for team effectiveness [57,58]. Furthermore, participation in decision-making is defined as joint decision-making [59] and involves sharing influence between team leaders and team members. By participating in decision-making, team members create work situations that are more favorable to their effectiveness. Team members utilize participating in decision-making for achieving what they desire for themselves and their team. Generally speaking, shared mental models are defined as organized knowledge structures that allow employees to interact successfully with their environment, and therefore lead to superior team performance [60]. That is, team members with a shared mental model about decision-making are ‘in sync’ and will easily coordinate their actions, whereas the absence of a shared mental model will result in process loss and ineffective team processes.

Taken together and based on the previous reasoning, we formulate the second hypothesis as follows:

Hypothesis 2: *Team resources (performance feedback*, *trust in management*, *team communication*, *and participation in decision-making) mediate the relationship between T1 team member’s shared perceptions of engaging leadership and T2 team effectiveness*.

### Engaging leadership, team resources, and work engagement (cross-level)

Engaging leaders build team resources (see above). Or put differently, the team member’s shared perceptions of engaging leadership are positively related to team resources. Besides, we also assume that these team resources positively impact work engagement at the individual level. A plethora of research has shown that various job resources are positively related to work engagement, including feedback, trust, communication, and participation in decision- making (for a narrative overview see [61]; for a meta-analysis see [62,63]). Most research that found this positive relationship between job resources and work engagement used the Job-Demands Resources model [5] that assumes that job resources are inherently motivating because they enhance personal growth and development and are instrumental in achieving work goals. Typically, these resources are assessed as perceived by the individual employee. Yet, as we have seen above, perceptions of resources might also be shared amongst team members. It is plausible that these shared resources, which collectively constitute a supportive, collaborative team climate, positively impact employee’s individual work engagement. Teams that receive feedback, have trust in management, whose members amply interact and communicate, and participate in decision-making are likely to produce work engagement. This reasoning agrees with Schaufeli [64], who showed that organizational growth climate is positively associated with work engagement, also after controlling for personality. When employee growth is deemed relevant by the organization this is likely to translate, via engaging leaders, into a supportive team environment, which provides feedback, trust, communication, and participative decision-making. Hence, we formulate:

Hypothesis 3: *Team resources (performance feedback*, *trust in management*, *team communication*, *and participation in decision-making) mediate the relationship between team shared perceptions of engaging leadership at T1 and individual team member’s work engagement at T2*.

## Method

### Sample and procedure

In collaboration with the HR department, data were collected among all employees of a business unit of a large Dutch public service agency. This agency is responsible for the administration of unemployment benefits and work incapacitation claims, as well as for the rehabilitation and return to work of unemployed and incapacitated employees. A one-year time-lagged design was applied to minimize the likelihood of common method variance effects and to explore causal relationships among the study variables [65]. The questionnaire included a cover letter reporting the aims and contents of the study. The letter also stated that participation in the study was completely voluntary, and that one can withdraw from the study at any time without having to give explanations and without this involving any disadvantage or prejudice. Participants’ consent was concluded by conduct, through ticking the consent checkbox as a prerequisite to access the questionnaire. This research was conducted in 2015, thus before the publication of the General Data Protection Regulation (GDPR) and complied with the latest version of the Declaration of Helsinki. Thus, ethics approval was not compulsory, as per applicable institutional and national Dutch guidelines. Additionally, the current study did not involve any treatment, medical diagnostics, or procedures generating psychological or social discomfort among participants.

In the first survey at *Time 1* (*N* = 2,304; response rate 63%), employees were asked about their socio-demographic background, engaging leadership, team resources (i.e., performance feedback, trust in management, communication, and participation in decision-making), team effectiveness, personal resources (i.e., resiliency, optimism, and flexibility), and work engagement. At *Time 2* (*N* = 2,183; response rate 51%), participants filled out the same survey, which included an additional self-efficacy scale. At both measurement points, participants received an email from the HR department containing a link that allowed them to fill out the online survey. This introductory email provided background information about the study’s general aim and guaranteed that participants’ responses would be treated confidentiality. A sample of *N* = 1,048 employees filled out the questionnaire twice, with an interval of one year between T1 and T2.

The estimation of multilevel models with at least 50 teams of at least 5 members per group is strongly recommended to avoid underestimating standard errors and variances for random effects [66,67]. Therefore, participants being part of teams with less than 5 employees were excluded from the analyses. Accordingly, the data of 1,048 participants, who completed both questionnaires, could be linked and constitute the current study sample. Employees were nested within 90 work teams, with an average of 13.7 (*SD* = 5.72) employees per team. Slightly more women (51.8%) as men were included (48.2%), the average age of the sample was 49.70 years (*SD* = 7.46), and the mean organization tenure was 12.02 years (*SD* = 9.56).

### Measures

All measures described below were rated using five-point scales that either ranged from *strongly disagree* (1) to *strongly agree* (5), or from *never* (1) to *always* (5). The internal consistencies (Cronbach’s α) of the measures are displayed on the diagonal of Table 2.

*Engaging Leadership* was measured using a scale developed by Schaufeli [64] including nine items. This questionnaire contains three subscales of three items each: Inspiring, Strengthening, and Connecting. Sample items are: “My supervisor is able to enthuse others for his/her plans” (inspiring); “My supervisor delegates tasks and responsibilities” (strengthening); and “My supervisor encourages team members to cooperate” (connecting).

#### Individual-level measures

*Optimism* was measured with three items from the Optimism scale of the PsyCap Questionnaire developed by Luthans and colleagues [36], which is aimed at assessing employees’ expectations about future success at work because of a positive view of their job. A sample item is: “I always look on the bright side of things regarding my job”.

*Resiliency* was assessed using three items from the Resiliency scale of the PsyCap Questionnaire [36]. These items refer to employees’ beliefs about their ability to recover from uncertainty and failure and to react successfully to setbacks that can occur at work. A sample item is: "I usually take stressful things at work in stride”.

*Self-efficacy* referred to the perceived capability to efficiently plan and implement courses of action required to attain a specific work goal and was measured using three items from Mazzetti, Schaufeli, and Guglielmi [68]. A sample item is: "At work, I reach my goal even when unexpected situations arise".

*Flexibility* refers to the individual ability to adapt to changes in the workplace and to modify one’s schedules and plans to meet job requirements. It was assessed by using three items: "If the job requires, I am willing to change my schedule”; “I do not have problems changing the way I work” and “I adapt easily to changes at work”.

*Work engagement* was assessed using a three-item scale developed by Schaufeli and colleagues [69]. This ultra-short version of the Utrecht Work Engagement Scale has similar psychometric properties as the nine-item version. A sample item is: "At my work, I feel bursting with energy”.

#### Team-level measures

*Performance feedback* was assessed by the three-item scale from the Questionnaire on the Experience and Evaluation of Work (QEEW) [70]. A sample item is: “Do you get enough information about the result of your work?”.

*Trust in Management* of team members was assessed using two items from Schaufeli [7]: “I trust the way my organization is managed”, and “I have confidence in my immediate supervisor”. Following the recommendations from Eisinga and colleagues [71] we computed the Spearman-Brown coefficient, since it represents the most appropriate reliability coefficient for a two-item scale (*r*_*s*_ = .43, *p* < .001).

*Communication*, meaning the perception of an efficient and prompt circulation of information at the team level was measured using the three-item Communication scale taken from the QEEW [70]. A sample item is: "I am sufficiently informed about the developments within my team”.

*Participation in decision-making* was measured by a single item (i.e., “Can you participate in decision making about work-related issues?”) from the QEEW [70].

*Team effectiveness*. The team-level criterion variable was assessed with a three-item scale [8]. A sample item is: “Do you cooperate effectively with others in your team?”.

#### Dropout

In order to check for systematic dropout, the social-demographic background, as well as the scores on the study variables were compared of those employees in the panel who filled out the questionnaire twice at T1 and T2 (*N* = 1,142) and those who dropped out and filled out the questionnaire only once at T1 (*N* = 1,161). It appeared that compared to the group who dropped out, the panel group was slightly younger (t_(2301)_ = -2.21; *p* < .05) and had less organizational tenure (t_(2301)_ = -4.05; *p* < .001). No gender differences were observed between both groups (χ^2^ = .88; *n*.*s*.). A multivariate analysis of variance (MANOVA) that included all study variables revealed a significant between-groups effect: F_(12,2291)_ = 3.54, *p* < .001. Subsequent univariate tests showed that compared to the dropouts, the panel group scored higher on inspiring (F_(1,2302)_ = 14.90, *p* < .001), strengthening (F_(1,2302)_ = 9.39, *p* < .01), and connecting leadership (F_(1,2302)_ = 14.90, *p* < .05), as well as on optimism (F_(1,2302)_ = 5.59, *p* < .05), flexibility (F_(1,2302)_ = 12.56, *p* < .001), work engagement (F_(1,2302)_ = 9.16, *p* < .05), performance feedback (F_(1,2302)_ = 11.68, *p* < .01), and participation in decision making (F_(1,2302)_ = 8.83, *p* < .05). No significant differences were found for resiliency, trust in management, communication, and team effectiveness.

It seems that, taken together, the panel group is slightly younger and less tenured, and scores more favorable than the dropouts on most study variables. However, the differences between both groups are relatively small and vary between 0 and .13 on a 5-point scale. Therefore, it is not likely that systematic dropout has influenced the results of the current study.

#### Control variables

At the individual level, we controlled for the potential confounding effects of gender, age, and tenure by including these variables as covariates in our analyses. More specifically, the impact of age was controlled for because previous research suggested that older employees report higher levels of personal resources [72] and work engagement [73]. Gender was also included as a control variable because previous research suggested that compared to women, men score lower on work engagement [74] and higher on personal resources, such as optimism and self-efficacy [75]. Finally, previous investigations also revealed that job tenure may affect employees’ level and stability of work engagement, with tenured employees reporting higher and more stable levels of work engagement compared to newcomers [76]. Besides, Barbier and colleagues [77] suggested that job tenure might affect employees’ personal resources (i.e., self-esteem and optimism). Considering this empirical evidence, job tenure was also included as a covariate in our model.

#### Data aggregation

Our research model includes the three dimensions of engaging leadership (i.e., inspiring, strengthening, and connecting) three team resources (i.e., performance feedback, trust in management, communication and participation in decision-making), and one outcome (i.e., team effectiveness) at the *team level* of analysis. To check the reliability and validity of aggregated scores at the team level, four indices were computed [78]: (1) ICC_[1]_, which indicates the proportion of variance in ratings due to team membership; (2) ICC_[2]_, representing the reliability of between-groups differences; (3) *r*_wg(j)_, that measures the level of agreement within work teams; (4) *deff*, that measures the effect of independence violations on the estimation of standard errors through the formula 1+(average cluster size-1)*ICC [79]. Generally speaking, values greater than .05 for ICC_[1]_ [80] and .40 for ICC_[2]_ [81] an *r*_wg(j)_ higher than .70, and a *deff-*index exceeding 2 are considered a prerequisite for aggregating data [78]. Moreover, one-way analysis of variance (ANOVA) was performed to explore whether participants’ scores on the Level 2 constructs differed significantly among work teams. The results of the aggregation tests are displayed in Table 1. Taken together, these results justify the aggregation of the team-level variables.

**Table 1:** Aggregation test results for team level variables.

Variables	Within-team agreement	Between-teams variance		Deff	Analysis of variance	
	*r*_wg(j)_	ICC_[1]_	ICC_[2]_		F(89, 958)	*p*
T1 Inspiring	.85	.16	.71	3.03	3.39	.000
T1 Strengthening	.90	.11	.61	2.40	2.60	.000
T1 Connecting	.87	.15	.68	2.91	3.11	.000
T2 Performance feedback	.85	.09	.55	2.14	2.22	.000
T2 Trust in management	.80	.16	.70	3.03	3.35	.000
T2 Communication	.86	.16	.71	3.03	3.41	.000
T2 Participation in decision-making	.65^a^	.17	.72	3.16	3.59	.000
T2 Team effectiveness	.83	.08	.51	2.02	2.02	.000

*Notes*. *N*_*individuals*_ = 1,048; *N*_*teams*_ = 90; Within-team agreement for measures with a single item is assessed using the *r*_wg_ index.

### Strategy of analysis

To test our hypotheses, a multilevel structural equation modeling (MSEM) was tested using the Mplus 7 statistical modeling software [82]. The application of this procedure allows the inclusion of latent variables that take measurement errors into account and permits the simultaneous estimation of mediation effects at the individual and team levels; therefore, it is superior to stepwise approaches [83]. As suggested by Zhang and colleagues [84], predictors at the individual level (i.e., engaging leadership dimensions and personal resources) were team-mean centered using a *centering within context*–*CWC* approach [85]. This procedure was aimed at preventing the confounding effect of mediation within and between work teams. In other words, predictors at the individual level for subject *i* were centered around the mean of the cluster *j* to which case *i* belongs (i.e., predictor_*ij*_—M_predictor*j*_). Accordingly, the latent engaging leadership factor at within-level was indicated by the CWC means of the three dimensions of engaging leadership (i.e., inspiring_*cwc*_, strengthening_*cwc*_, and connecting_*cwc*_) at T1. In a similar vein, personal resources were included as a latent variable indicated by the observed levels of optimism_*cwc*_, reisliency_*cwc*_, self-efficacy_*cwc*_, and flexibility_*cwc*_ at T2. Finally, T2 work engagement was included as an observed variable equal to the mean score of the corresponding scale. As previously stated, gender, age, and organizational tenure were included as covariates at the individual level of the MSEM model.

At the team level, the latent measure of engaging leadership at T1 was assessed through the observed scores on the three dimensions of inspiring, strengthening, and connecting leadership. T2 team resources were modeled as a single latent factor indicated by the observed scores on performance feedback, trust in management, communication, and participation in decision-making. The observed mean score on T2 team effectiveness was modeled as the team level criterion variable.

At the individual level, the mediation was tested by considering *path a*, from T1 individual perceptions of engaging leadership (X) to T2 personal resources (M) and *path b*, from T2 personal resources to T2 work engagement (Y), controlling for X → Y. At the team level, the same procedure was applied considering *path c*, linking team perceptions of T1 engaging leadership (X) and T2 team resources (M) and *path d*, from T2 team resources to T2 team effectiveness (Y).

The individual and team-level perceptions of engaging leadership were assessed at T1. In contrast, the mediating variables (i.e., psychological capital and team resources), and the outcomes (i.e., work engagement and team effectiveness) were measured at T2.

## Results

### Preliminary analysis

Before testing our hypotheses, a confirmatory factor analysis (CFA) was performed using the maximum likelihood method of estimation using the software package AMOS 21.0 [86]. This preliminary analysis was aimed at assessing redundancy between the constructs under investigation. For the team level, engaging leadership was included as a latent factor indicated by the observed team levels of inspiring, strengthening, and connecting leadership dimensions. The measured performance feedback levels indicated the latent team resources factor, trust in management, communication, and participation in decision-making. Team effectiveness, assessed as a criterion variable at the team level, was indicated by a single corresponding item. At the individual level, the group-mean centered scores on inspiring, strengthening, and connecting dimensions were considered indicators of the latent engaging leadership factor. Besides, optimism, resiliency, self-efficacy, and flexibility were included as indicators for the single personal resources latent factor; the observed average score on work engagement was used for assessing the corresponding latent variable. The model fit was assessed by considering the comparative fit index (CFI) and Tucker-Lewis Index (TLI) ≥ .95, Root-Mean-Square Error of Approximation (RMSEA) ≤ .06, and Standardized Root-Mean-Square Residual (SRMR) ≤ .08 [87,88]. According to these criteria, the hypothesized measurement model showed a good fit to the data, with χ^2^ (91) = 465.09, CFI = .96, TLI = .95, RMSEA = .06, and SRMR = .03. Moreover, all indicators showed significant factor loadings on their respective latent factors (*p* < .001) with λ values ranging from .51 to .95, thus exceeding the commonly accepted criterion of .50 [89]. Hence, these results support the assumption that the study variables were non-redundant and adequately distinct from each other.

### Model testing

The means, standard deviations, correlations, and internal consistencies for all study variables are displayed in Table 2. As expected, the constructs under investigation showed significant relationships in the hypothesized direction.

**Table 2:** Means, standard deviation, and zero-order correlations among the variables.

			*r*															
	*M*	*SD*	1	2	3	4	5	6	7	8	9	10	11	12	13	14	15	16
1. T1 Gender	.48	.50	—															
2. T1 Age	49.7	7.96	.32***	—														
3. T1 Organizational tenure	12.02	9.56	.02	.37***	—													
4. T1 Inspiring	3.48	.79	-.07*	.00	-.03	(.89)												
5. T1 Strengthening	3.91	.62	-.02	.01	-.03	.67***	(.80)											
6. T1Connecting	3.61	.72	-.02	.00	-.02	.73***	.66***	(.86)										
7. T2 Optimism	3.55	.76	.02	.05	.00	.19***	.21***	.20***	(.81)									
8. T2 Resilience	3.95	.53	.06*	.05	-.02	.10**	.15***	.12***	.47***	(.82)								
9. T2 Self-efficacy	3.72	.65	.10**	.04	-.01	.12***	.17***	.14***	.50***	.51***	(.85)							
10. T2 Flexibility	3.92	.59	.02	-.02	-.09**	.16***	.21***	.16***	.44***	.49***	.43***	(.81)						
11. T2 Work engagement	3.4	.74	-.04	.09**	.00	.27***	.28***	.26***	.54***	.33***	.41***	.38***	(.75)					
12. T2 Performance feedback	2.55	.69	.10**	.01	-.02	.30***	.29***	.28***	.25***	.19***	.26***	.18***	.30***	(.76)				
13. T2 Trust in management	3.37	.73	.00	.00	-.03	.37***	.32***	.34***	.29***	.12***	.14***	.20***	.29***	.40***	—			
14. T2 Communication	3.24	.65	.09**	.10**	.00	.30***	.31***	.32***	.28***	.14***	.22***	.18***	.26***	.40***	.57***	(.68)		
15. T2 Participation in decision-making	3.01	.92	.07*	.01	-.07*	.29***	.29***	.27***	.27***	.20***	.22***	.24***	.33***	.36***	.40***	.41***	—	
16. T2 Team effectiveness	3.64	.75	-.04	.06	.11**	.20***	.24***	.26***	.27***	.17***	.25***	.19***	.34***	.26***	.25***	.32***	.21***	(.82)

*Notes*. *N*_*individuals*_ = 1,048; *N*_*teams*_ = 90; Gender was coded as 0 = female and 1 = male.

*p < .05

**p < .01

***p < .001. Scale reliabilities (Cronbach’s alpha) are in parentheses along the diagonal.

**Fig 2:**
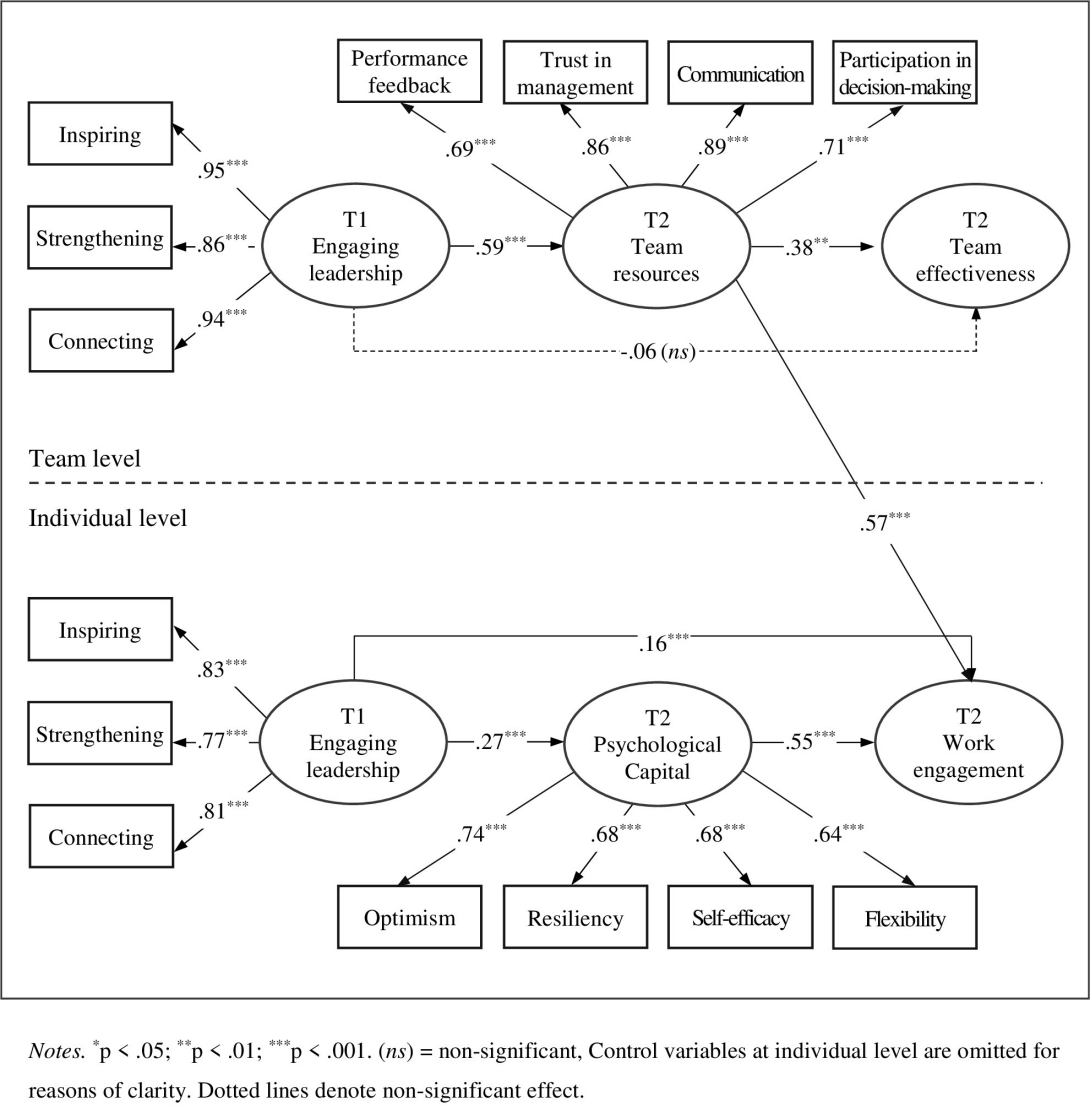
Standardized path coefficients of the final model.

The hypothesized MSEM showed a good fit to data: χ^2^(60) = 155.38, CFI = .97, TLI = .96, RMSEA = .04, SRMR = 0.03 (within teams) and .08 (between teams). As displayed in Fig 2, at the individual level the three indicators of engaging leadership loaded significantly on their intended latent factor, with λ = .83 (*p* = .000, 95% CI = [.79, .87]) for inspiring, λ = .77 (*p* < .001, 95% CI = [.73, .81]) for strengthening, and λ = .81 (*p* < .001, 95% CI = [.78, .85]) for connecting. Similarly, the standardized factor loadings for the indicators of personal resources were all significant as well: λ = .74 (*p* < .001, 95% CI = [.68, .79]) for optimism, λ = .68 (*p* < .001, 95% CI = [.63, .72]) for resiliency λ = .68 (*p* < .001, 95% CI = [.62, .74]) for self-efficacy, and λ = .64 (*p* < .001, 95% CI = [.59, .69]) for flexibility.

The direct relationship between T1 engaging leadership and T2 work engagement was significant β = .16 (*p* < .001, 95% CI = [.10, .22]). Moreover, results indicated that engaging leadership at T1 had a positive impact on personal resources at T2: γ = .27 (*p* < .001, 95% CI = [.18, .37]). T2 personal resources, in turn, were positively associated with T2 work engagement: β = .55 (*p* < .001, 95% CI = [.49, .62]). The estimated indirect effect of T1 engaging leadership on T2 work engagement via personal resources (i.e., a*b) was statistically significant: B (SE) = .19 (.04), *p* < .001, 95% CI [.11, .27]. Hence, personal resources (i.e., optimism, resiliency, self-efficacy, and flexibility) at T2 partially mediated the impact of T1 engaging leadership on employees’ engagement within work teams at T2. These findings provide partial support for *Hypothesis 1*. Among the covariates included at the individual level, only gender and age showed a significant association with work engagement, with γ = -.10 (*p* < .001, 95% CI = [-.15, -.05]) and γ = .10 (*p* < .001, 95% CI = [.04, .16]), respectively.

At the team level, all factor loadings for the three indicators of engaging leadership on their corresponding latent variable were significant: λ = .95 (*p* < .001, 95% CI = [.93, .99]) for inspiring, λ = .86 (*p* < .001, 95% CI = [.80, .91]) for strengthening, and λ = .94 (*p* < .001, 95% CI = [.90, .97]) for connecting. Additionally, the observed measure of each team resource loaded significantly on its intended latent variable: λ = .69 (*p* < .001, 95% CI = [.56, .82]) for performance feedback, λ = .86 (*p* < .001, 95% CI = [.78, .94]) for trust in management, λ = .89 (*p* < .001, 95% CI = [.81, .97]) for communication, and λ = .71 (*p* = .000, 95% CI = [.60, .82]) for participation in decision-making. Moreover, engaging leadership at T1 had a nonsignificant direct impact on team effectiveness at T2, with β = -.06 (*p* = .641, 95% CI = [-.30, .19]). In contrast, team perception of engaging leadership at T1 had a positive impact on team resources at T2: γ = .59 (*p* < .001, 95% CI = [.42, .75]). Team resources at T2 were, in turn, positively related to T2 team effectiveness, β = .38 (*p* = .003, 95% CI = [.13, .62]). These results suggest full mediation and were supported by the estimation of the indirect effect of T1 engaging leadership on T2 team effectiveness via team resources at T2 (i.e., c*d): B (SE) = .18 (.07), *p* = .013, 95% CI [.04, .32]. Thus, *Hypothesis 2* was fully supported.

Hence, in the current study team resources at T2 (i.e., performance feedback, trust in management, communication, and participation in decision-making) fully mediated the effect of T1 engaging leadership on T2 team effectiveness across different work teams. Moreover, T2 team resources (i.e., performance feedback, trust in management, team communication, and participation in decision-making) showed a significant cross-level effect on T2 individual team member’s level of engagement: β = .57 (p < .001, 195% CI = [.27, .87]). This result provided evidence for *Hypothesis 3*.

## Discussion

The current study aimed to explore the role of individual and collective perceptions of engaging leadership in predicting team effectivity and work engagement. To this purpose, we developed a two-level research model using a two time-point design.

### Main results

At the individual level, the obtained results suggest that psychological capital (i.e., the combination of self-efficacy, optimism, resiliency, and flexibility) partly mediated the longitudinal relationship between employees’ perceptions of engaging leadership and their levels of work engagement. In other words, team leaders perceived as inspiring, strengthening, and connecting could enhance their followers’ engagement directly and indirectly through an increase in psychological capital. Thus, engaging leaders could make their followers feel more optimistic, resilient, self-efficacious, and flexible. At the team level, a shared perception of engaging leadership was associated with a greater pool of team resources (i.e., performance feedback, trust in management, communication, and participation in decision-making), which contribute to define an open and supportive team climate that is conducive for employee growth and development. In their turn, these collective resources were positively related to the perceived effectiveness of work teams.

Hence, team resources at the team level fully mediated the relationship between engaging leadership and team effectiveness. That means that teams in which the leader is considered to be inspiring, strengthening, and connecting can draw upon more team resources, and could feel, in turn, more effective. Simultaneously, a significant *cross-level* mediation effect was found for team resources, meaning that they mediate the relationship between engaging leadership at team level and individual level work engagement. In other words, teams with engaging leaders are not only more effective at the team level, but they also report higher levels of work engagement among their members. These leaders create a team climate that fosters employee growth and development by providing performance feedback, installing trust, and stimulating communication and participation in decision-making.

#### Three different contributions

Thus, three major conclusions can be drawn for the current study, which signifies its contribution to the literature. First, engaging leadership can be considered an individual-level construct (i.e., the perception of particular leadership behaviors by individual followers) and a collective, team-level construct (i.e., the shared perception of specific leadership behaviors among team members). As far as the latter is concerned, our results support the notion of an average leadership style (ALS) [47]; namely, that homogeneous leader-follower interactions exist *within* teams, but relationships of leaders with followers differ *between* teams.

Secondly, Individual-level engaging leadership predicts individual work engagement through increasing follower’s PsyCap. Previous research suggested a positive relationship between person-focused leadership styles and follower’s work engagement, albeit that virtually all studies were cross-sectional in nature (for an overview see [11,33]). Our study added longitudinal evidence for that relationship and hinted at an underlying psychological process by suggesting that psychological capital might play a mediating role. As such, the current study corroborates and extends a previous cross-sectional study that obtained similar results [8]. However, it should be noted that the present study used a slightly different operationalization of PsyCap as is usually employed [36]. In addition to the three core elements of optimism, resiliency, and self-efficacy, flexibility instead of hope was used as a constituting fourth element of PsyCap. The reason was that hope and optimism overlap both theoretically as well as empirically [35] and that flexibility–defined as the ability to readapt, divert from unsuccessful paths, and tackle unpredictable conditions that hinder employees’ goal attainment [8]–was deemed particularly relevant for public service agencies that are plagued by red tape. Our results indicate that engaging leaders strengthen followers’ sense of proficiency when developing a task-specific skill to reach challenging objectives (i.e., self-efficacy). They also encourage a favorable appraisal of the prevailing conditions and future goal achievement (i.e., optimism).

Furthermore, they enhance subordinates’ abilities to recover from failures and move beyond setbacks effectively (i.e., resiliency) through supporting an increased aptitude for adaption to unfamiliar work circumstances (i.e., flexibility). These results corroborate the assumption that leaders who inspire, strengthen, and connect their followers provide a stimulating work environment that enhances employees’ personal resources. In their turn, elevated levels of PsyCap mobilize employees’ energy and intrinsic motivation to perform, expressed by a high level of work engagement. This result concurs with previous evidence that PsyCap can be framed as a critical component of the motivational process of the JD-R model, namely as a mediator of the relationship between contextual resources (i.e., engaging leadership) and work engagement [46]. However, this mediation was only partial because a direct effect of engaging leadership on follower’s work engagement was also observed in the current study. This evidence is not surprising since previous research showed that other mediating factors (which were not included in the present study) played a role in explaining the relationship between leadership and work engagement. Among them, innovative work behaviors, meaningful work, role clarity, positive emotions, identification with the organization, and psychological ownership [11]. Thus, increasing their follower’s PsyCap is not the whole story as far as the impact of engaging leadership is concerned. It is likely that engaging leaders also impact these alternative mediating factors. If this is the case, this might explain why the additional variance in follower’s work engagement is explained by engaging leadership, as indicated by the direct effect.

Thirdly, team-level engaging leadership predicts work engagement of individual team members and team effectiveness through increasing team resources. An earlier cross-sectional study found that engaging leadership, as perceived by their followers, showed an indirect, positive effect on their work engagement level through an increase in job resources [7]. However, in that study, engaging leadership and job resources, including performance feedback, trust in management, communication, and participation in decision-making, were assessed at the individual and not at the aggregated team level. This means that the current study corroborates previous findings at the aggregated team level, using a longitudinal design. It is important to note that employees’ level of work engagement not only depends on individual-level processes (through the increase in PsyCap) but also on collective processes (trough the rise in team resources). Finally, our findings concur with research on team climate, showing that leaders who endorse supportive relations between team members and create an open, empowering team climate enable employees to succeed [33]. Simultaneously, a team climate like that is also likely to foster personal growth and development, which, in turn, translates into greater work engagement [63].

### Practical implications

Our study shows that engaging leadership matters, and therefore organizations are well-advised to stimulate their managers to lead by the principles of engaging leadership. To that end, organizations may implement leadership development programs [90], leadership coaching [91], or leadership workshops [92]. Previous research has shown that leadership behaviors are malleable and subject to change using professional training [93–95]. Furthermore, leaders may want to establish and promote an open and trusting team climate in which employees feel free to express their needs and preferences [96,97].

Accordingly, our study shows that this climate is conducive not only for work engagement but also for team effectiveness. Finally, our results also suggest that psychological capital is positively associated with work engagement, so that it would make sense to increase this personal resource, mainly because PsyCap is state-like and open to development through instructional programs [45]. For instance, a short PsyCap Intervention (PCI) has been developed by Luthans and colleagues, which is also available as a web-based version for employees [98]. PCI focuses on: (a) acquiring and modifying self–efficacy beliefs; (b) developing realistic, constructive, and accurate beliefs; (c) designing goals, pathway generation, and strategies for overcoming obstacles; and (d) identifying risk factors, and positively influencing processes.

### Strengths, limitations, and directions for future research

A significant strength of the current study is its design that combines a multilevel investigation of engaging leadership with mediating processes at the individual and team levels. This is in line with the claim that leadership research suffers from a lack of theoretical and empirical differentiation between levels of analysis [99]. However, leadership is an inherently multilevel construct in nature [9]. Although the current findings shed light on the role of the emergent construct of engaging leadership, both regarding individuals and teams, an exciting venue for future research involves exploring its predictive validity in comparison with traditional leadership models. This concurrent validation would adhere to the recommendations accompanying the introduction of new leadership constructs in the face of the risk of construct proliferation [16].

A further strength of the current study is its large sample size, including 1,048 employees nested within 90 work teams. Moreover, data were collected at two time points with a one-year time lag that was considered long enough for the effects of engaging leadership to occur. In contrast with widespread cross-sectional studies that sometimes draw unjustified conclusions on the corollaries of leadership [100], the current research relied on a longitudinal design to better understand the consequences of engaging leadership at the individual and team level of analysis. According to our results, engaging leadership indeed shows a positive effect across time on outcomes at the individual (i.e., work engagement) and team level (i.e., team effectiveness).

Along with its strengths, the current study also has some limitations that should be acknowledged. The main weakness of the current study lies in the homogeneity of the sample, which consisted of employees working in a Dutch public service agency. This specific work setting prevents us from generalizing the findings of our research with other occupational groups. However, focusing on an organization where most activities are conducted in teams permits independent but simultaneous assessment of the impact of (engaging) leadership on the perceived pool of resources among teams and workers, as suggested by current trends in leadership literature [101,102].

Furthermore, the collection of data at different time points overcomes the inherent weakness of a cross-sectional design, yet a design including at least three data waves would have provided superior support for the hypothesized mediated relationships. Based on within-group diary studies [103,104], it can, on the one hand, be argued that leadership might impact the team and personal resources within a much shorter time frame. On the other hand, work engagement represents a persistent psychological state that is not susceptible to sudden changes in the short term [1]. Thus, the chosen one-year time lag can be considered reasonable for a between-group study to detect the impact of engaging leadership accurately. This impact needs some time to unfold. An additional limitation of this study entails measuring individual and team resources with only a few items. Nevertheless, all scales had an internal consistency value that met the threshold of .65 [105] with an average Cronbach’s alpha value equal to .81.

## Concluding remark

Despite the novelty of the construct, the emerging research on engaging leadership suggests the potential value of a theoretically sound leadership model that could foster followers’ engagement. While earlier findings showed that engaging leadership is positively *associated* with the employee’s level of engagement [7,8], the current study suggested that engaging leadership *could predict* work engagement and team effectiveness. More specifically, being exposed to a leader who inspires, strengthens and connects team members may foster a shared perception of greater availability of team resources (i.e., performance feedback, trust in management, communication, and participation in decision-making), as well as greater psychological capital (i.e., self-efficacy, optimism, resilience, and flexibility). Hence, engaging leadership could play a significant role in the processes leading to work engagement at both the team and the individual levels.

## Supporting information

S1 Datset (XLSX)

## References

[1] Schaufeli W.B., Salanova M., González-Romá V., Bakker A.B. The measurement of engagement and burnout: A two sample confirmatory factor analytic approach. Journal of Happiness Studies. 2002; 3(1), 71–92. https://doi.org10.1023/A:1015630930326.

[2] Björk J.M., Bolander P., Forsman A.K. Bottom-up interventions effective in promoting work engagement: A Systematic review and meta-analysis. Frontiers in Psychology. 2021; 3754. doi: 10.3389/fpsyg.2021.730421 34566819 PMC8456101

[3] Mazzetti G., Robledo E., Vignoli M., Topa G., Guglielmi D., Schaufeli W.B. Work engagement: A meta-analysis using the Job Demands-Resources model. Psychological Reports. 2021; 1–38. doi: 10.1177/00332941211051988 34886729

[4] Schaufeli W.B. Applying the Job Demands-Resources model: A ‘how to’ guide to measuring and tackling work engagement and burnout. Organizational Dynamics. 2017; 46(2), 120–132. 10.1016/j.orgdyn.2017.04.008.

[5] Bakker A.B., Demerouti E. The job demands-resources model: State of the art. Journal of Managerial Psychology. 2007; 22(3), 309–328. 10.1108/02683940710733115.

[6] Deci E.L., Ryan R.M. The" what" and" why" of goal pursuits: Human needs and the self-determination of behavior. Psychological Inquiry. 2000; 11(4), 227–268. 10.1207/S15327965PLI1104_01.

[7] Schaufeli W.B. Engaging leadership in the job demands-resources model. Career Development International. 2015a. 20(5), 446–463. 10.1108/CDI-02-2015-0025.

[8] Schaufeli W.B. Van burnout naar bevlogenheid Werk en welbevinden in Nederland [From burnout to work engagement: Work and well-being in the Netherlands]. M&O. 2015b; 69, pp.15–31.

[9] Bliese P.D., Halverson R.R., Schriesheim C.A. Benchmarking multilevel methods in leadership: The articles, the model, and the data set. The Leadership Quarterly 2002; 13(1), 3–14. 10.1016/S1048-9843(01)00101-1.

[10] Yukl G. Managerial leadership: A review of theory and research. Journal of management. 1989;15(2), 251–289. 10.1177/014920638901500207.

[11] Carasco-Saul M., Kim W., Kim T. Leadership and employee engagement: Proposing research agendas through a review of literature. Human Resource Development Review. 2015; 14(1), 38–63. 10.1177/1534484314560406.

[12] DeCuypere A., Schaufeli W.B. Leadership and work engagement: Exploring explanatory mechanisms. German Journal of Human Resource Management. 2019; 1–27. https://doi.org/2397002219892197.

[13] Antonakis J., Day D.V. Leadership: Past present and future. In, Antonakis J. & Day D.V.(Eds.), The nature of leadership (3rd. Ed.) London: Sage. 2017; 3–28.

[14] Van Knippenberg D., Sitkin S.B. A critical assessment of charismatic-transformational leadership research: Back to the drawing board?. The Academy of Management Annals. 2013; 7(1), 1–60. 10.1080/19416520.2013.759433.

[15] Deci E.L., Ryan R.M. Self-determination theory. In Van Lange P.A.M., Kruglanski A.W., & Higgins E.T.(Eds.), Handbook of theories of social psychology. London: Sage. 2012; 416–436. 10.4135/9781446249215.n21.

[16] Bormann K.C., Rowold J. Construct proliferation in leadership style research: Reviewing pro and contra arguments. Organizational Psychology Review. 2018; 8(2–3), 149–173. 10.1177/2041386618794821.

[17] Erasmus, A. (2018). Investigating the relationships between engaging leadership, need satisfaction, work engagement and workplace boredom within the South African mining industry (Doctoral dissertation, North-West University).

[18] Robijn W., Euwema M.C., Schaufeli W.B., Deprez J. Leaders, teams and work engagement: a basic needs perspective. Career Development International. 2020; 25(4), 373–388. 10.1108/CDI-06-2019-0150.

[19] Van Tuin L., Schaufeli W.B., Van Rhenen W. The satisfaction and frustration of basic psychological needs in engaging leadership. Journal of Leadership Studies. 2020a; 14(2), 6–23. 10.1002/jls.21695.

[20] Rahmadani V.G., Schaufeli W.B., Ivanova T.Y., Osin E.N. Basic psychological need satisfaction mediates the relationship between engaging leadership and work engagement: A cross‐national study. Human Resource Development Quarterly. 2019; 30(4), 453–471. 10.1002/hrdq.21366.

[21] Robijn, W. (2021). “Taking care of the team member is taking care of the team: a team conflict perspective on engaging leadership,” in: Leadership and Work Engagement: A Conflict Management Perspective, ed W. Robijn (Unpublished PhD) (Belgium: KU Leuven), 79–107.

[22] Rahmadani V.G., Schaufeli W.B., Stouten J. How engaging leaders foster employees’ work engagement. Leadership & Organization Development Journal. 2020a; 41(8), 1155–1169. 10.1108/LODJ-01-2020-0014.

[23] Nikolova I., Schaufeli W.B., Notelaers G. Engaging leader–Engaged employees? A cross-lagged study on employee engagement. European Management Journal. 2019; 37(6), 772–783. 10.1016/j.emj.2019.02.004.

[24] Van Tuin L., Schaufeli W.B., Van den Broeck A. Engaging leadership: Enhancing work engagement through intrinsic values and need satisfaction. Human Resource Development Quarterly. 2021; 32(4), 483–505. 10.1002/hrdq.21430.

[25] Salas-Vallina A., Alegre J., Lopez-Cabrales A. The challenge of increasing employees’ wellbeing and performance: How human resource management practices and engaging leadership work together toward reaching this goal. Human Resource Management. 2021; 60(3), 337–347. 10.1002/hrm.22021.

[26] Rahmadani V.G., Schaufeli W.B., Stouten J., Zhang Z., Zulkarnain Z. Engaging leadership and its implication for work engagement and job outcomes at the individual and team level: A multi-level longitudinal study. International Journal of Environmental Research and Public Health. 2020b; 17(3), 776. doi: 10.3390/ijerph17030776 31991897 PMC7037879

[27] Van Tuin L., Schaufeli W.B., Van Rhenen W., Kuiper R.M. Business results and well-being: An engaging leadership intervention study. International Journal of Environmental Research and Public Health. 2020b; 17(12), 4515. doi: 10.3390/ijerph17124515 32585988 PMC7345976

[28] Mäkikangas A., Bakker A.B., Schaufeli W.B. Antecedents of daily team job crafting. European Journal of Work and Organizational Psychology. 2017; 26(3), 421–433. 10.1080/1359432X.2017.1289920.

[29] Yukl G., Gordon A., Taber T. A hierarchical taxonomy of leadership behavior: Integrating a half century of behavior research. Journal of Leadership & Organizational Studies. 2002; 9(1), 15–32. doi: 10.1177/107179190200900102

[30] Bass B.M., Riggio R.E. Transformational leadership. In *Transformational leadership*. 2nd ed. Psychology Press Ltd., 2006.

[31] Smith, D.J. (2018). The Importance of Self-Determined Leadership in Building Respectful Workplace. (Unpublished PhD thesis), University of Southern Queensland, Australia.

[32] Rahmadani V.G., Schaufeli W.B. Engaging leadership and work engagement as moderated by “diuwongke”: an Indonesian study. The International Journal of Human Resource Management. 2020c; 1–29. 10.1080/09585192.2020.1799234.

[33] Serrano S.A., Reichard R.J. Leadership strategies for an engaged workforce. Consulting Psychology Journal: Practice and Research. 2011; 63(3), 176–189. 10.1037/a0025621.

[34] Luthans F., Youssef C.M., Avolio B.J. Psychological capital: Investing and developing positive organizational behaviour. In Nelson D & Cooper C. L.(Eds.), Positive organizational behaviour. London: Sage. 2007; pp. 9–24.

[35] Ouweneel E., Le Blanc P.M., Schaufeli W.B. Don’t leave your heart at home: Gain cycles of positive emotions, resources, and engagement at work. Career Development International. 2012; 17(6), 537–556. 10.1108/13620431211280123.

[36] Luthans F., Avolio B.J., Avey J.B., Norman S.M. Positive psychological capital: Measurement and relationship with performance and satisfaction. Personnel Psychology. 2007; 60(3), 541–572. https://doi.org/j.1744-6570.2007.00083.x.

[37] Luthans F., Avey J.B., Avolio B.J., Norman S.M., Combs G.M. Psychological capital development: toward a micro‐intervention. Journal of Organizational Behavior: The International Journal of Industrial, Occupational and Organizational Psychology and Behavior. 2016; 27(3), 387–393. 10.1002/job.373.

[38] Gooty J., Gavin M., Johnson P.D., Frazier M.L., Snow D.B. In the eyes of the beholder: Transformational leadership, positive psychological capital, and performance. Journal of Leadership & Organizational Studies. 2009; 15(4), 353–367. 10.1177/1548051809332021.

[39] Rego A., Sousa F., Marques C., Cunha M. Authentic leadership promoting employees’ psychological capital and creativity. Journal of Business Research. 2012; 65(3), 429–437. 10.116/j.jbusres.2011.10.003.

[40] Bandura A. Cultivate self-efficacy for personal and organizational effectiveness. In Locke E. (Ed.), Handbook of principles of organizational behavior. Oxford: Blackwell. 2000; pp. 120–136.

[41] Sweetman D., Luthans F. The power of positive psychology: Psychological capital and work engagement. In Bakker A.B. & Leiter M. P. (Eds.), Work engagement: A handbook of essential theory and research. New York: Psychology Press. 2010; pp. 44–68.

[42] Fredrickson B.L. Positive emotions broaden and build. In Devine P., & Plant A. (Eds.), Advances in Experimental Social Psychology (ed., Vol. 47). San Diego, CA: Academic Press. 2013; pp. 1–54.

[43] Luthans F., Youssef C.M. Emerging positive organizational behavior. Journal of Management. 2007; 33(3), 321–349. 10.1177/0149206307300814.

[44] Paek S., Schuckert M., Kim T.T., Lee G. Why is hospitality employees’ psychological capital important? The effects of psychological capital on work engagement and employee morale. International Journal of Hospitality Management. 2015; 50, 9–26. 10.1016/j.ijhm.2015.07.001.

[45] Luthans F., Youssef-Morgan C.M. Psychological capital: An evidence-based positive approach. Annual Review of Organizational Psychology and Organizational Behavior. 2017; 4, 339–366. 10.1146/annurev-orgpsych-032516-113324.

[46] Mazzetti G., Guglielmi D., Chiesa R., Mariani M.G. Happy employees in a resourceful workplace: just a direct relationship? A study on the mediational role of Psychological Capital. Career Development International. 2016; 21(7), 682–696. 10.1108/CDI-03-2016-0035.

[47] Yammarino F.J., Bass B.M. Transformational leadership and multiple levels of analysis. Human Relations. 1990; 43(10), 975–995. 10.1177/001872679004301003.

[48] Bass B.M. Leadership and performance beyond expectation. New York: Free Press; 1985.

[49] Burke C.S., Stagl K.C., Klein C., Goodwin G.F., Salas E., Halpin S.M. What type of leadership behaviors are functional in teams? A meta-analysis. The Leadership Quarterly. 2006; 17(3), 288–307. 10.1016/j.leaqua.2006.02.007.

[50] Ceri-Booms M., Curşeu P.L., Oerlemans L.A. Task and person-focused leadership behaviors and team performance: A meta-analysis. Human Resource Management Review. 2017; 27(1), 178–192. 10.1016/j.hrmr.2016.09.010.

[51] McGrath J.E. Social psychology: A brief introduction. New York: Holt, Rinehart and Winston; 1964.

[52] DeShon R.P., Kozlowski S.W., Schmidt A.M., Milner K.R., Wiechmann D. A multiple-goal, multilevel model of feedback effects on the regulation of individual and team performance. Journal of Applied Psychology. 2004; 89(6), 1035–1056. doi: 10.1037/0021-9010.89.6.1035 15584840

[53] Mayer R.C., Davis J.H., Schoorman F.D. An integrative model of organizational trust. Academy of Management Review. 1995; 20(3), 709–734. 10.5465/amr.1995.9508080335.

[54] Braun S., Peus C., Weisweiler S., Frey D. Transformational leadership, job satisfaction, and team performance: A multilevel mediation model of trust. The Leadership Quarterly. 2013; 24(1), 270–283. 10.1016/j.leaqua.2012.11.006.

[55] Arnold K.A. Transformational leadership and employee psychological well-being: A review and directions for future research. Journal of Occupational Health Psychology. 2017; 22(3), 381–393. doi: 10.1037/ocp0000062 28150998

[56] Ilgen D.R., Hollenbeck J.R., Johnson M., Jundt D. Teams in organizations: From input-process-output models to IMOI models. Annual Review of Psychology. 2005; 56(1), 517–543. doi: 10.1146/annurev.psych.56.091103.070250 15709945

[57] Barry B., Stewart G.L. Composition, process, and performance in self-managed groups: The role of personality. Journal of Applied Psychology. 1997; 82(1), 62–78. 10.1037/0021-9010.82.1.62. 9119798

[58] Hyatt D.E., Ruddy T.M. An examination of the relationship between work group characteristics and performance: Once more into the breech. Personnel Psychology. 1997; 50(3), 553–585. 10.1111/j.1744-6570.1997.tb00703.x.

[59] Wagner J.A. III, Leana C.R., Locke E.A., Schweiger D.M. Cognitive and motivational frameworks in U.S. research on participation: A meta-analysis of primary effects. Journal of Organizational Behavior. 1997; 18(1), 49–65. https://doi.org./10.1002/(SICI)1099-1379(199701)18:1<49::AID-JOB789>3.0.CO;2-P.

[60] Mathieu J.E., Heffner T.S., Goodwin G.F., Salas E., Cannon-Bowers J.A. The influence of shared mental models on team process and performance. Journal of Applied Psychology. 2000; 85(2), 273–283. doi: 10.1037/0021-9010.85.2.273 10783543

[61] Schaufeli W.B. What is engagement? In Employee engagement in theory and practice. Routledge. London: Routledge. 2013; pp. 29–49.

[62] Crawford E.R., LePine J.A., Rich B.L. Linking job demands and resources to employee engagement and burnout: a theoretical extension and meta-analytic test. Journal of Applied Psychology. 2010; 95(5), 834–848. doi: 10.1037/a0019364 20836586

[63] Goering D.D., Shimazu A., Zhou F., Wada T., Sakai R. Not if, but how they differ: A meta-analytic test of the nomological networks of burnout and engagement. Burnout Research; 2017; 5, 21–34. 10.1016/j.burn.2017.05.003.

[64] Schaufeli W.B. Heavy work investment, personality and organizational climate. Journal of Managerial Psychology; 2016; 31(6), 1057–1073. 10.1108/JMP-07-2015-0259.

[65] Podsakoff P.M., MacKenzie S.B., Lee J.Y., Podsakoff N.P. Common method biases in behavioral research: A critical review of the literature and recommended remedies. Journal of Applied Psychology; 2003; 88(5), 879–903. doi: 10.1037/0021-9010.88.5.879 14516251

[66] Hox J.J. Multilevel analyses: Techniques and applications. Mahwah, NJ: Erlbaum; 2002.

[67] Hox J.J. Multilevel analysis: Techniques and applications (2nd ed.). New York, NY: Routledge; 2010.

[68] Mazzetti G., Schaufeli W.B., Guglielmi D. Are workaholics born or made? Relations of workaholism with person characteristics and overwork climate. International Journal of Stress Management. 2014; 21(3), 227–254. 10.1037/a0035700.

[69] Schaufeli W.B., Shimazu A., Hakanen J., Salanova M., De Witte H. An ultra-short measure for work engagement: The UWES-3 validation across five countries. European Journal of Psychological Assessment. 2017; 35(4), 577–591. 10.1027/1015-5759/a000430.

[70] Van Veldhoven M.V., Jonge J.D., Broersen S., Kompier M., Meijman T. Specific relationships between psychosocial job conditions and job-related stress: A three-level analytic approach. Work & Stress. 2002; 16(3), 207–228. 10.1080/02678370210166399.

[71] Eisinga R., Grotenhuis M.T., Pelzer B. The reliability of a two-item scale: Pearson, Cronbach, or Spearman-Brown? International Journal of Public Health. 2013; 58(4), 637–642. 10.1007/s00038-012-0416-3. 23089674

[72] Martínez-Martí M.L., Ruch W. Character strengths predict resilience over and above positive affect, self-efficacy, optimism, social support, self-esteem, and life satisfaction. The Journal of Positive Psychology. 2017; 12(2), 110–119. 10.1080/17439760.2016.1163403.

[73] Demerouti E., Bakker A.B., Gevers J.M. Job crafting and extra-role behavior: The role of work engagement and flourishing. Journal of Vocational Behavior. 2015; 91, 87–96. 10.1016/j.jvb.2015.09.001.

[74] Harju L.K., Hakanen J.J., Schaufeli W.B. Can job crafting reduce job boredom and increase work engagement? A three-year cross-lagged panel study. Journal of Vocational Behavior. 2016; 95, 11–20. 10.1016/j.jvb.2016.07.001.

[75] Kocalevent R.D., Klapp B.F., Albani C., Brähler E. Gender differences in a resources-demands model in the general population. BMC Public Health. 2014; 14(1), 902–909. 10.1186/1471-2458-14-902. 25178159 PMC4246563

[76] Agarwal U.A. Linking justice trust and innovative work behavior to work engagement. Personnel Review; 2013; 43(1), 41–73. 10.1108/PR-02-2012-0019.

[77] Barbier M., Hansez I., Chmiel N., Demerouti E. Performance expectations, personal resources, and job resources: How do they predict work engagement? European Journal of Work and Organizational Psychology. 2013; 22(6), 750–762. 10.1080/1359432X.2012.704675.

[78] Bliese P.D. Within-group agreement, non-independence, and reliability: Implications for data aggregation and analysis. In Klein K. J. & Kozlowski S. W. J. (Eds.), Multilevel theory, research, and methods in organizations: Foundations, extensions, and new directions. San Francisco, CA: Jossey-Bass; 2000, pp. 349–381.

[79] Muthén B., Satorra A. Complex sample data in structural equation modeling. In Marsden P. V. (Ed.), Sociological methodology. Washington, DC: American Sociological Association; 1995, pp. 264–316. doi: 10.2307/271070

[80] LeBreton J.M., Senter J.L. Answers to 20 questions about interrater reliability and interrater agreement. Organizational Research Methods. 2007;11(4), 815–852. 10.1177/1094428106296642.

[81] Glick W. Conceptualizing and measuring organizational and psychological climate: Pitfalls in multilevel research. Academy of Management Review. 1985; 10, 601–616. 10.5465/AMR.1985.4279045.

[82] Muthén B.O., Muthén L.K. Mplus version 7 [Computer Programme]. Los Angeles, CA: Muthén & Muthén; 2012.

[83] Preacher K.J., Zyphur M.J., Zhang Z. A general multilevel SEM framework for assessing multilevel mediation. Psychological Methods. 2010; 15(3), 209–233. doi: 10.1037/a0020141 20822249

[84] Zhang Z., Zyphur M.J., Preacher K.J. Testing multilevel mediation using hierarchical linear models: Problems and solutions. Organizational Research Methods. 2009; 12(4), 695–719. 10.1177/1094428108327450.

[85] Kreft I.G., De Leeuw J., Aiken L.S. The effect of different forms of centering in hierarchical linear models. Multivariate Behavioral Research. 1995; 30(1), 1–21. doi: 10.1207/s15327906mbr3001_1 26828342

[86] Arbuckle J.L. Amos (Version 21.0). Chicago: IBM SPSS; 2015.

[87] Brown T. Confirmatory factor analysis for applied research. New York, NY: Guilford Press; 2006.

[88] Hu L., Bentler P.M. Cutoff criteria for fit indexes in covariance structure analysis: Conventional criteria versus new alternatives. Structural Equation Modeling. 1999; 6(1), 1–55. 10.1080/10705519909540118.

[89] Hair J.F., Black W.C., Babin B.J., Anderson R.E., Tatham R.L. Multivariate Data Analysis. Uppersaddle River, NJ: Prentice-Hal Hall; 2006.

[90] Shuck B., Herd A.M. Employee engagement and leadership: Exploring the convergence of two frameworks and implications for leadership development in HRD. Human Resource Development Review. 2012; 11(2), 156–181. 10.1177/1534484312438211.

[91] Ely K., Boyce L.A., Nelson J.K., Zaccaro S.J., Hernez-Broome G., Whyman W. Evaluating leadership coaching: A review and integrated framework. The Leadership Quarterly. 2010; 21(4), 585–599. 10.1016/j.leaqua.2010.06.00355.

[92] Segers J., De Prins P., Brouwers S. Leadership and engagement: A brief review of the literature, a proposed model, and practical implications. In Albrecht S. L. (Ed.), New horizons in management. Handbook of employee engagement: Perspectives, issues, research and practice. Edward Elgar Publishing; 2010, pp. 149–158.

[93] Nielsen K., Randall R., Christensen K.B. Does training managers enhance the effects of implementing team-working? A longitudinal, mixed methods field study. Human Relations. 2010; 63(11), 1719–1741. 10.1177/0018726710365004.

[94] Jackson R.J., Lindsay D.R. Lessons for experience: Why wait? Industrial and Organizational Psychology: Perspectives on Science and Practice. 2010; 3(1), 48–51. 10.1111/j.1754-9434.2009.01197.x.

[95] Parry K.W., Sinha P.N. Researching the trainability of transformational organizational leadership. Human Resource Development International. 2005; 8(2), pp. 165–183. 10.1080/13678860500100186.

[96] Burke C.S., Sims D.E., Lazzara E.H., Salas E. Trust in leadership: A multi-level review and integration. The Leadership Quarterly; 2007;18(6), 606–632. 10.1016/j.leaqua.2007.09.006.

[97] Schaufeli W.B. Engaging leadership: How to promote work engagement?. Frontiers in Psychology. 2021; 12. doi: 10.3389/fpsyg.2021.754556 34777155 PMC8578900

[98] Luthans F., Avey J.B., Patera J.L. Experimental analysis of a web–based training intervention to develop positive psychological capital. Academy of Management Learning & Education. 2008; 7(2), 209–221. 10.5465/AMLE.2008.32712618.

[99] Yammarino F.J., Dionne S.D., Chun J.U., Dansereau F. Leadership and levels of analysis: A state-of-the-science review. The Leadership Quarterly. 2005; 16(6), 879–919. 10.1016/j.leaqua.2005.09.002.

[100] Ployhart R.E., Holtz B.C., Bliese P.D. Longitudinal data analysis: Applications of random coefficient modeling to leadership research. The Leadership Quarterly. 2002; 13(4), 455–486. 10.1016/S1048-9843(02)00122-4.

[101] Day D.V., Gronn P, Salas E. Leadership in team-based organizations: On the threshold of a new era. The Leadership Quarterly. 2006; 17(3), 211–216. 10.1016/j.leaqua.2006.02.001.

[102] Morgeson F.P., DeRue D.S., Karam E.P. Leadership in teams: A functional approach to understanding leadership structures and processes. Journal of Management. 2010; 36(1), 5–39. 10.1177/0149206309347376.

[103] Breevaart K., Bakker A.B. Daily job demands and employee work engagement: The role of daily transformational leadership behavior. Journal of Occupational Health Psychology. 2018; 23(3), 338–349. doi: 10.1037/ocp0000082 28358569

[104] Tims M., Bakker A.B., Xanthopoulou D. Do transformational leaders enhance their followers’ daily work engagement?. The Leadership Quarterly. 2011; 22(1), pp.121–131. 10.1016/j.leaqua.2010.12.011.

[105] DeVellis R.F. Scale development: Theory and applications (Vol. 26). London: Sage publications; 2016.

